# Double Narrow Fano Resonances via Diffraction Coupling of Magnetic Plasmon Resonances in Embedded 3D Metamaterials for High-Quality Sensing

**DOI:** 10.3390/nano11123361

**Published:** 2021-12-11

**Authors:** Haitao Hu, Xue Lu, Jianhua Huang, Kai Chen, Jun Su, Zhendong Yan, Chaojun Tang, Pingen Cai

**Affiliations:** 1 Department of Physical Education, Nanjing Forestry University, Nanjing 210037, China; hhtshell@163.com; 2 Collaborative Innovation Center of Atmospheric Environment and Equipment Technology, Jiangsu Key Laboratory of Atmospheric Environment Monitoring and Pollution Control (AEMPC), Nanjing University of Information Science & Technology, Nanjing 210044, China; xlu@njfu.edu.cn (X.L.); catqchen@163.com (K.C.); 3 College of Science, Nanjing Forestry University, Nanjing 210037, China; jhhuang@njfu.edu.cn (J.H.); sujun@njfu.edu.cn (J.S.); zdyan@njfu.edu.cn (Z.Y.); 4 Center for Optics and Optoelectronics Research, Collaborative Innovation Center for Information Technology in Biological and Medical Physics, College of Science, Zhejiang University of Technology, Hangzhou 310023, China

**Keywords:** magnetic plasmon resonances, fano resonance, split-ring resonators, metamaterial, sport doping

## Abstract

We theoretically demonstrate an approach to generate the double narrow Fano resonances via diffraction coupling of magnetic plasmon (MP) resonances by embedding 3D metamaterials composed of vertical Au U-shaped split-ring resonators (VSRRs) array into a dielectric substrate. Our strategy offers a homogeneous background allowing strong coupling between the MP resonances of VSRRs and the two surface collective optical modes of a periodic array resulting from Wood anomaly, which leads to two narrow hybridized MP modes from the visible to near-infrared regions. In addition, the interaction effects in the VSRRs with various geometric parameters are also systematically studied. Owing to the narrow hybrid MP mode being highly sensitive to small changes in the surrounding media, the sensitivity and the figure of merit (*FoM*) of the embedded 3D metamaterials with fabrication feasibility were as high as 590 nm/RIU and 104, respectively, which holds practical applications in label-free biosensing, such as the detection of medical diagnoses and sport doping drugs.

## 1. Introduction

Plasmonic nanostructures and metamaterials have drawn considerable attention due to their remarkable optical properties resulting from the collective oscillations of free electrons, named as surface plasmons (SP) [1,2,3,4]. The localized SP resonances can strongly enhance the localized electromagnetic fields and are in high demand for high sensitivity detection of the specific molecules, which could be used in food safety, medical diagnoses and sport doping drugs monitoring [5,6,7,8,9]. Recently, metamaterial composed of subwavelength metallic split-ring resonators (SRRs) has attracted much interest due to their extraordinary optical properties and potential applications, particularly in magnetic nonlinearity and magnetic sensors [10,11,12,13,14,15,16,17,18,19,20].

So far, most reported SRRs are placed directly on the top surface of a dielectric substrate. Owing to radiative or absorptive loss, however, the magnetic plasmon (MP) resonances usually have a broad linewidth and largely leaks into the substrates to decrease the effective sensing volume and further limit the sensing performance [21,22]. In order to solve this problem, the three-dimensional (3D) metamaterials composed of the vertical SRR (VSRR) array have been recently reported by Tsai’s group [23,24,25,26]. For these 3D metamaterials, the VSRR arrays lifted off from the substrates are standing on substrates, causing the magnetic fields to be localized in the VSRR gaps, which is fully exposed to the sensing medium [27]. Because of the superior optical field distribution properties, the metamaterials consisting of VSRRs have been utilized as high-quality optical sensors [28].

On the other hand, it is important to narrow the bandwidth of the MPs of SRRs to improve the sensing performance. An effective method to achieve the narrow bandwidth of MP resonances is to couple the broad MP mode of SRRs with other optical modes with a narrow bandwidth through near-field localized plasmon hybridization [29,30,31] or diffraction coupling [32,33,34,35] in the periodic nanoparticle array to generate the MP-based Fano resonance effect [36,37]. It is well known that Fano resonances exhibit the electromagnetic response with the characteristic asymmetric narrow lineshapes, which arises from the near-field coupling between the two transition channels, i.e., a plasmonic dark mode and a bright mode [38,39,40]. For the diffraction coupling in the VSRR structure, a narrow-band hybrid MP mode with an asymmetric Fano lineshape is generated by the coupling between a broad MP mode and a collective optical mode propagated along the surface in a periodic array [35]. However, due to the practical nanoparticle arrays usually being fabricated on top surface of a substrate, for the collective optical modes propagating above and below the substrate, the refraction index mismatch would lead to different phase velocities and conditions for constructive interference [41]. Therefore, considering the fabrication feasibility and the practical applications, it is necessary to satisfy the homogeneous dielectric environment of conventional particle arrays to obtain the asymmetric narrow Fano resonances.

In this work, we propose an effective approach to achieve the ultranarrow double narrow Fano resonances in visible and near-infrared ranges in the 3D metamaterial consisting of Au VSRRs array via the diffraction coupling of MP resonances. Through embedding the periodic arrays of VSRRs into the substrate, strong diffraction coupling between the MP resonance of an individual VSRRs and two collective surface modes of the periodic array propagated in air and in the silica substrate generate the two narrow-band hybridized MP modes. The coupling effects of the geometrical parameters on the double narrow Fano resonances are also systematically studied. Our designed 3D metamaterials with fabrication feasibility exhibits a high sensitivity (*S* = 590 nm/RIU) and figure of merit (*FoM* = 104), which is a good candidate for label-free detection of medical diagnoses and sport doping drugs.

## 2. Materials and Methods

**Figure 1:**
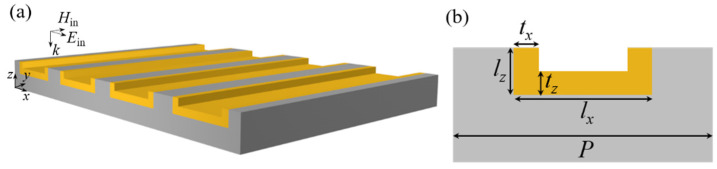
(**a**) Schematic view of the 3D Au U-shaped VSRRs array embedded in a silica substrate. (**b**) The magnified front views of a unit cell of the Au VSRRs array. The arm length and base length of the VSRR, the Au thickness along the *x* and *z* axis, and the period of the VSRR array are denoted as *l*_z_, *l*_x_, *t*_x_, *t*_z_, and *P*, respectively.

Figure 1a schematically shows the structures of the proposed Au VSRRs array embedded in the silica substrate. The incident light with transverse magnetic (TM) polarization is also shown. In Figure 1b, the magnified front view of a unit cell of the Au VSRRs array embedded in a silica substrate is depicted. The arm length and base length of the VSRR, the Au thickness along the *x* and *z* axis, and the period of the VSRR array are denoted as *l*_z_, *l*_x_, *t*_x_, *t*_z_, and *P*, respectively. The length of Au VSRR along the *y* axis is infinite. Numerical calculation is performed by the software package “EastFDTD, version 5.0” based on the finite difference time domain method. Periodic boundary conditions are used along the *x*-axis, and two perfectly matched layers (PML) are set along the the *z*-axis to eliminate the boundary scattering of the electromagnetic waves, respectively. The Gauss pulse is set to be a light source. The transmission spectra are calculated by Fourier transform and the electric and magnetic field distributions can be simulated directly. The minimum mesh size of Au VSRR is set to be 4 nm. For the other region, the homogeneous mesh size is set to be Δs = 20 nm, and the corresponding time step Δt is expressed as Δs/2c, where c is light speed in a vacuum. The permittivity of Au was calculated by using the Drude model with the plasma frequency of *ω*_p_ = 1.37 × 1016 s^−1^ and the damping constant of *ω*_c_ = 4.08 × 1013 s^−1^ [42]. The refractive index (*n*_d_) of the silica substrate is 1.45. The designed 3D metamaterials embedded in a dielectric substrate can be realized in a lab as follows: Firstly, masking by the photoresist, the U-shaped groove is transferred to the silica substrate by the ICP dry-etching. During ICP dry-etching process, the fluorinated plasma gas is used and the chamber pressure is kept at 3.5 Pa. Then, our proposed Au U-shaped VSRRs could be realized by Au evaporation and lift off process. During the Au film deposition process, a thin Au film (t = 20 nm) was deposited onto the silica substrate in a vacuum of 5 × 10^−^^6^ Torr at a rate of 0.5 A˚/s by cathode ion sputtering method (IBC Model 682, Gatan Corp., Pleasanton, CA, USA). We used a tape to lift off the Au film directly on the top surface of the dielectric substrate to obtain the proposed Au U-shaped VSRRs array. These mentioned fabrication processes have been reported in our previous work [43,44]. It is worth noting that, unlike the U-shaped VSRRs on substrate proposed by other experimental research groups, our designed Au U-shaped VSRRs embedded in a substrate could be easily fabricated without the costly and time-consumed second electron beam lithography method [21].

## 3. Results and Discussion

**Figure 2:**
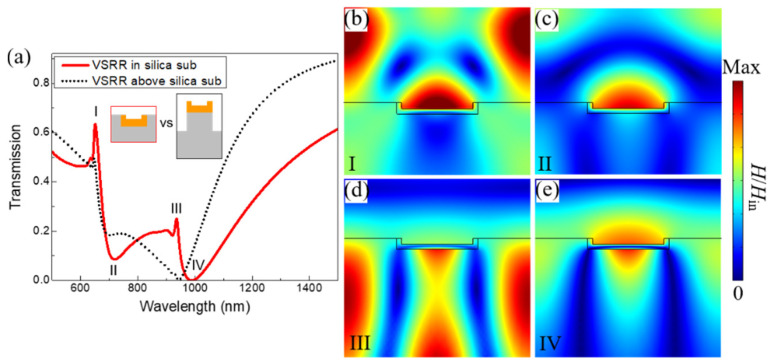
(**a**) The calculated transmission of Au VSRR array embedded in the silica substrate and Au VSRR array suspended by silica pillars. The inset schematically shows the magnified view of the two metamaterials. (**b**–**e**) Normalized magnetic field (*H*/*H*_in_) distributions of Au VSRR array embedded in the silica substrate from the front view at the corresponding transmission resonances of I, II, III and IV, respectively. The structural sizes of the Au VSRR array embedded in a silica substrate: *t*_x_ = *t*_z_ = 20 nm, *l*_x_ = 290 nm, *l*_z_ = 40 nm, and *P* = 650 nm, respectively.

Figure 2 presents the plasmonic properties of the Au VSRRs array embedded in a silica substrate under the normal incidence of light for TM polarization. The geometric sizes of the Au VSRR array embedded in a silica substrate: *t*_x_ = *t*_z_ = 20 nm, *l*_x_ = 290 nm, *l*_z_ = 40 nm, and *P* = 650 nm, respectively. As shown by the red solid line in Figure 2a, for the embedded Au VSRRs array, there are two clear, narrow Fano transmission peaks at 650.5 nm and 935.6 nm, denoted as peak I and III. The two narrow bandwidths were 12.4 nm and 9.7 nm for the two corresponding Fano transmission peaks I and III, respectively. Here, the bandwidth is expressed by the full width at half maximum of the spectrum (*FWHM*). The broad transmission dips II and IV represent the fundamental MP resonance of an individual VSRR. The two relatively narrow Fano transmission peaks I and III are due to the two hybrid MP modes resulting from the diffraction coupling between the broad bright MP resonances of the individual SRRs and the two narrow dark in-plane collective surface modes propagated in air and in the silica substrate, which is further discussed below in Figure 2b–e. The positions of the two in-plane collective surface modes propagated in air and in the silica substrate under normal incidence are expressed as *λ*_I_ = *mP* and *λ*_III_ = *mn*_d_*P* [33], where *λ* is the resonant wavelength, *m* is the integer of the different diffraction orders, *n*_d_ is the refractive index of the silica substrate, *P* is the period. Here, *m*, *n*_d_ and *P* are 1, 1.45 and 650 nm, respectively. Then, the two resonant positions of *λ*_I_ and *λ*_III_ were calculated to be 650 nm and 942.5 nm, which correspond to the positions of the two narrow Fano transmission peaks I and III shown in Figure 1a.

As we all know, the Fano resonance of metallic nanoparticle arrays fabricated on substrates is weak due to the refraction index mismatch. Therefore, in practice, there are two approaches to reduce the refraction index mismatch to enhance the Fano resonance caused by diffraction coupling. One approach is to suspend the metallic nanoparticle arrays to reduce the effect of the substrate [34]. The other is to embed metallic nanoparticle arrays into the substrate, which is proposed in our manuscript. For comparison, we calculated the transmittance of the Au VSRRs array suspended by the silica nanopillars on the silica substrate with the same structural sizes of an individual Au VSRR and the same period shown by the black dotted line. Here, the height of silica nanopillar is 100 nm. It can be seen that there are two differences for the array suspended by the silica nanopillars on the dielectric substrate comparing to the transmittance of the embedded one. One difference is the disappearance of the Fano resonance at the peak III. The other difference is the intensity of the hybridized MP mode at the Fano peak I becoming weaker. Both the two differences result from the inefficient diffraction coupling due to the inhomogeneous environment. Auguié group have also investigated the influence of a homogeneous dielectric environment on the surrounded nanoparticles in order to obtain a clear single narrow Fano resonance as a result of effective diffraction coupling [45]. Here, it should be noted that our designed embedded 3D VSRRs array is not in a fully homogeneous dielectric environment with a large difference of the refractive index between the layer of the dielectric substrate and the upper layer of the VSRR array. In this case, double narrow Fano resonances through diffraction coupling of magnetic plasmon resonances in our designed embedded 3D metamaterials of the Au VSRR array were obtained.

To further get a deeper insight into the underlying physics for the double Fano resonance excitations in the 3D embedded Au VSRRs arrays, the normalized magnetic field distributions from front view for the two narrow Fano transmission peaks I, III and the corresponding two broad transmission dips II, IV of the designed 3D metamaterials are plotted from Figure 2b to Figure 2e. For the broad transmission dips II and IV, the magnetic fields are mainly localized at the Au VSRR surface, which represents the pure MP resonances of an individual Au VSRRs [21]. Meanwhile, at the transmission peaks I and III, it can be clearly seen that the two collective surface modes propagating in the air and in the silica substrate are both excited simultaneously in our designed embedded 3D metamaterials.

**Figure 3:**
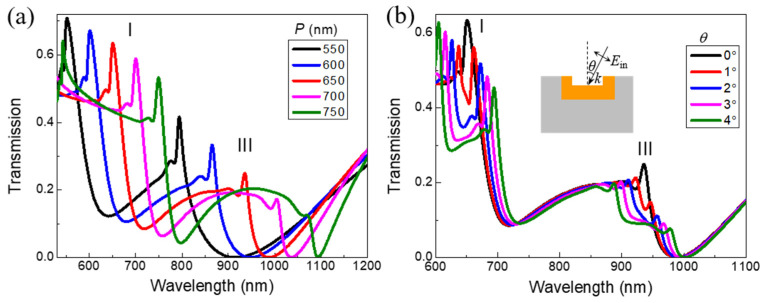
(**a**) The calculated transmission spectra of the Au VSRR embedded in the silica substrate for different period (*P*) under normal incidence of light for TM polarization, where the other geometric parameters are the same as shown in Figure 2a. (**b**) The calculated transmission spectra of the Au VSRR embedded in the silica substrate for various incident angles (*θ*) with TM polarization, where all the geometric parameters are the same as shown in Figure 2a.

Figure 3 shows the transmission response to different periods (*P*) of the array of our designed embedded 3D metamaterials and incident angle (*θ*). As shown in Figure 3a, as *P* increases from 550 nm to 750 nm in steps of 50 nm, a pronounced red-shift of the double narrow Fano resonances at peak I and III under normal incidence is observed when *P* is steadily increased. Engineering on the period can be performed to tune the double Fano resonances of the proposed embedded 3D metamaterials from visible to near-infrared range. Figure 3b shows that for oblique light incidence, when the incident angle (*θ*) slightly increases from 1° to 4° in steps of 1°, both the narrow Fano transmission peaks (I and III) are split to two new narrower peaks compared to those under normal incidence (*θ* = 0°). Such new splitting modes under oblique light incidence with small incident angle decrease radiative losses and further narrow the linewidth to make the quality factor higher for oblique light incidence than that for the normal light incidence.

**Figure 4:**
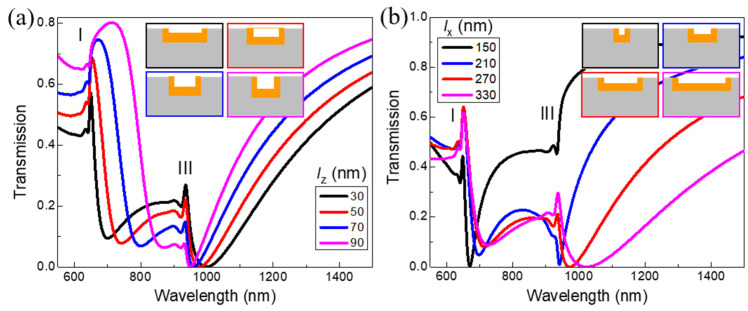
(**a**) The calculated transmission spectra of the Au VSRRs array embedded in the silica substrate for different *l*_z_ under normal incidence of light for TM polarization. The whole length (2*l*_z_+ *l*_x_) of VSRR, *t*_x_, *t*_z_ and *P* are kept as 370 nm, 20 nm, 20 nm and 650 nm, respectively. (**b**) The calculated absorption spectra for different *l*_x_ under normal incidence of light for TM polarization. Here, *l*_z_, *t*_z_, *t*_x_ and *P* are kept as 40 nm, 20 nm, 20 nm and 650 nm, respectively.

Then we investigate the effects of the geometric parameters of the arm length (*l*_z_) and the base length (*l*_x_) on the transmission of the Au VSRR embedded in the silica substrate under normal-incidence. Figure 4a shows the calculated transmission spectra of the embedded Au VSRR for different *l*_z_ under normal incidence of light for TM polarization. Here, we keep 2*l*_z_ + *l*_x_ = 370 nm, *t*_z_ = *t*_x_ = 20 nm, and *P* = 650 nm. The inset of Figure 4a shows the corresponding four magnified schematic views of Au VSRR embedded in the silica substrate with different *l*_z_. Figure 4a shows that the linewidth of peak I becomes broader and the intensity of peak III decreases as the arm length *l*_z_ increases from 30 nm to 90 nm in intervals of 20 nm. The double narrow Fano resonance modes were clearly obtained when the structural parameter of the arm length (*l*_z_) is set at 30 nm. Figure 4b shows that the intensities of both the narrow peaks I and III were increased as the base length *l*_x_ is changed from 150 nm to 330 nm in steps of 60 nm. Here, *l*_z_, *t*_z_, *t*_x_ and *P* were set as 40 nm, 20 nm, 20 nm and 650 nm, respectively. The inset of Figure 4a shows the corresponding four magnified schematic views of Au VSRR embedded in the silica substrate with different *l*_x_.

**Figure 5:**
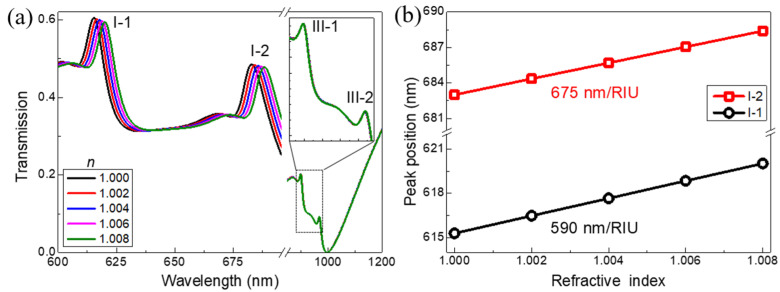
(**a**) The calculated transmission spectra of the designed 3D metamaterials with inclined incidence angle (*θ*) being 3° immersed in surrounding medium varied from 1.000 to 1.008. (**b**) The dependence of the positions of two peaks (I-1 and I-2) as a function of refractive index.

Finally, we investigate the performance of our proposed 3D metamaterials on sensing delicate variations in the refractive index (RI) of the ambient perturbations. The sensitivity (*S*) and figure of merit (*FoM*) are evaluated by the expressions: *S* = Δ*λ*/Δ*n*, *FoM* = *S*/*FWHW* [28]. Here, Δ*λ* is the spectral shift as a result of the RI change Δ*n*. The transmission spectra of the designed metamaterials immersed in different surrounding environments were calculated with an inclined incidence angle (*θ*) of 3°. The geometric parameters were kept the same as those shown in Figure 2a. In Figure 5a, as the RI increases from 1.000 to 1.008 in steps of 0.002, the spectral red-shift of the double transmission peaks (I-1 and I-2) were observed. The *FWHW* of the two transmission peaks I-1 and I-2 were 5.7 nm and 7.9 nm, respectively. However, the transmission spectra of the two transmission peaks (III-1 and III-2) were almost not varied for the different RI of the surrounding media, which is owing to the fact that the hybridized MP modes were excited inside the substrate as shown in Figure 2d.

The positions of the two transmission peaks (I-1 and I-2) as a function of the RI of the surrounding media is shown in Figure 5b. By the linearly fitting, the sensitivities *S* of the two narrow two transmission peaks I-1 and I-2 were found to be 590 nm/RIU and 675 nm/RIU, respectively. The *FoM* values were 104 for transmission peak I-1 and 85 for the transmission peak I-2. The high sensitivity (*S*) and figure of merit (*FoM*) for the narrowband hybridized mode at the transmission peaks (I-1) were now nearly among the highest values of the recently reported plasmonic sensors [46], which could be used to detect the small changes of the RI of different environment media. Recently, there has been an increasing interest in the high-pressure plasmonic sensing under elevated pressure conditions [47,48,49]. Owing to the high value of the sensitivity and *FoM*, our proposed 3D matamaterials could further be a plasmonic optical pressure sensor. In addition, due to the fabrication feasibility, our proposed 3D metamaterials have practical applications in label-free detection of medical diagnoses and sport doping drugs.

## 4. Conclusions

In conclusion, we have demonstrated the double narrow Fano resonances are realized in the 3D Au VSRRs array embedded into the dielectric substrate, via the diffraction coupling between the magnetic fundamental plasmon resonance of an individual VSRR and two collective surface modes resulting from Wood anomaly in the periodic array. The clearly excited double narrow Fano resonances are attributed to the more homogeneous background in our proposed 3D metamaterials. The coupling effects of the structural parameters and incident light angle on the double narrow Fano resonances were also studied. The proposed 3D metamaterials fabricated by Au evaporation and lift off process with fabrication feasibility exhibit a high sensitivity (*S* = 590 nm/RIU) and figure of merit (*FoM* = 104), which hold practical applications in label-free detection of sport doping drugs and medical diagnoses.

## Data Availability

The data presented in this study are available on request from the first or corresponding author.
